# Optical and Electrical Analyses of Solar Cells with a Radial PN Junction and Incorporating an Innovative NW Design That Mimics ARC Layers

**DOI:** 10.3390/nano13101649

**Published:** 2023-05-16

**Authors:** Francisco J. Cabrera-España, B. M. Azizur Rahman

**Affiliations:** School of Science and Technology, City, University of London, London EC1V0HB, UK; francisco.cabrera@city.ac.uk

**Keywords:** nanowires, solar cell, light trapping, texturing patterns, cost reduction, absorption enhancement, radial pn junction

## Abstract

The implementation of a texturing pattern on the surface of a solar cell is well known for reducing reflection, thus increasing the absorption of sunlight by the solar cell. Nanowires (NWs) that are large in their height have been widely used for this purpose. Through rigorous numerical simulations, this work explores the benefits of short but index-matched NWs and how these designs are also affected by surface recombination. Additionally, this work further optimized power conversion efficiency (PCE) by placing two or three NWs of different heights and diameters on top of each other to mimic the performance of two-NW and three-NW ARC designs with PCEs of 16.8% and 17.55%, respectively, when a radial pn junction is considered. These are the highest reported so far for such a thin silicon solar cell. Furthermore, we also show how these designs were impacted by surface recombination velocity and compare these findings to simple NWs of different heights and diameters.

## 1. Introduction

The generation of energy from renewable sources has experienced a rapid increase in recent years. This has been driven by the awareness of policymakers and of society in general about the importance of minimizing some of the catastrophic effects of climate change [1,2,3,4,5,6]. Nevertheless, these reports also indicate that most of the energy consumed nowadays is still generated by burning fossil fuels, leading to significant CO_2_ emissions [7]. This suggests that there is still a need for improvement to encourage the faster deployment of renewable energy devices such as photovoltaic (PV) solar cells. In any case, it is encouraging that these reports also indicate that PV solar cells have already made a large contribution toward the efforts for cleaner energy generation [8]. Sunlight is abundant, available to everyone, and free to access, and the current level of the technology involved offers a range of products and very promising performance [9]. Solar cells have experienced constant improvement toward more efficient absorption of sunlight as well as more efficient internal extraction of electrons, leading to higher power conversion efficiency (PCE). For instance, the milestone of 26.5% PCE under lab conditions was obtained in June 2022 for a traditional single junction using a thick wafer (at least 165 µm) of crystalline silicon solar cell [10,11]. Furthermore, newer types of PV solar cell technologies, such as perovskite-based solar cells, have emerged and are receiving a lot of attention [12]. Although perovskite-based solar cells have recently shown significant improvements in their PCE values, their reliability issues and concerns about potential lead (Pb) leakage to the environment still need to be resolved [12]. However, there is, of course, room for further improvement regarding the key aspects of Si solar cell technology and increasing the number of PV solar cells deployed, as well as reducing the amount of energy-intensive silicon required for the fabrication of highly efficient PV solar cells [13], energy storage (for its availability at all the times) [14], internal recombination losses [15,16], effects of moisture [17], shading [18,19], temperature [20], and the Schockley-Queisser limit [21,22].

The absorption of sunlight can be enhanced by innovations like concentrated solar cells, anti-reflection coating (ARC) layers, and textured surfaces, taking advantage of light trapping. The latter consist of placing an array of regular geometrical patterns, such as pyramids [23], micropillars [24], nanorods [25], nanowires (NW) [26], or nanoholes (NH) [27]. By doing this, the incoming sunlight incident on the solar cell surface bounces multiple times between the elements of the array, thus enhancing absorption [23,24,26,28]. Moreover, it is important to highlight that the high level of absorption obtained when using surface texturing techniques, such as NWs, enables the use of thin substrates of lower quality silicon when compared to the typical 200–400 µm thick low-defect (requiring a large amount of energy consumption, which increases cost) silicon substrate required for the fabrication of traditional crystalline silicon solar cells [10,13,29,30]. Nevertheless, there is a different perspective with which to view this NW array as a layer of refractive index between that of silicon and air [31]. Therefore, instead of the sunlight being incident on a single silicon–air interface with a large refractive index difference, by introducing two interfaces with two smaller refractive index differences and following Fresnel’s equation, the sunlight reflected at these two interfaces (air-textured pattern and textured-patterned solar cell) is considerably reduced, thus increasing the sunlight absorption.

## 2. Results and Discussions

**Figure 1:**
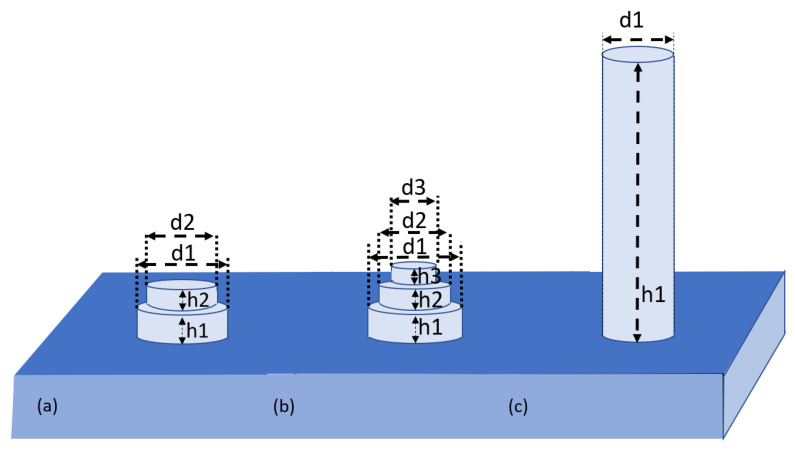
Schematic diagram (**a**) of the two-NW ARC design; (**b**) the three-NW ARC design; (**c**) simple NW.

In our earlier work [32], we rigorously evaluated the performance of the often-ignored short NWs by using the finite difference time domain (FDTD) method [33] incorporated in the Lumerical FDTD-solutions package [34]. We have shown that NWs with a height of as low as 130 nm offer the same total absorption (TABS) when integrated over the solar spectrum as NWs that are as high as 1450 nm, significantly reducing the usage of silicon. The short 130 nm high NW mimics the performance of a single-layered ARC layer with an ideal refractive index and minimizes the reflectance between the air and silicon (i.e., geometric average between the two refractive indices), where the refractive index can be adjusted by varying the diameter of the NW. Thereafter, we have used this concept and optimized it further by placing two or three NWs of different diameters and heights on top of each other, as illustrated in Figure 1a,b, to mimic the performance of a two- or three-layered ARC design.

**Figure 2:**
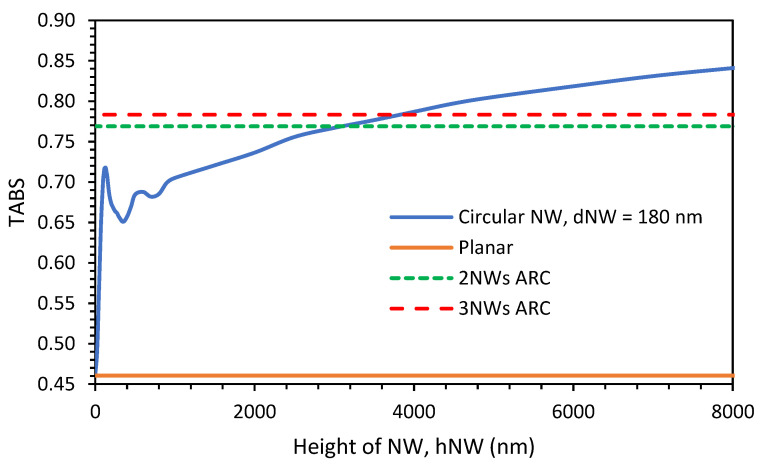
Variations in TABS as hNW increases when compared to when using two-NW and three-NW ARC design TABS.

The variations in TABS with the height of the NW are shown by a solid blue line in Figure 2 for a simple NW (diameter: 180 nm). This diameter yields the equivalent index of the layer, satisfying anti-reflection coating functionality. The value of TABS for the case of a planar surface is shown by a solid orange line as a reference. From Figure 2, it can be observed that an NW that is 130 nm in height has the same TABS value as that of an NW with a height of 1450 nm. The value of TABS for the already optimized two-NW (d1 = 254 nm, d2 = 150 nm, h1 = 130 nm, and h2 = 90 nm with total height 220 nm) and three-NW (d1 = 272 nm, d2 = 203 nm, d3 = 116 nm, h1 = 91 nm, h2 = 88 nm, and h3 = 89 nm, with a total height of 268 nm) ARC designs are shown by dashed green and dashed red lines, respectively.

From Figure 2, the benefits of the two-NW and three-NW ARC designs can be clearly observed when compared to simple NWs. In the case of the two-NW ARC (total height = 220 nm), the value of TABS (0.769) is equivalent to that of an NW of 3450 nm in height, and in the case of the three-NW ARC design (total height = 268 nm), the value of TABS (0.784) is equivalent to that of an NW of 4270 nm in height. This clearly demonstrates that high absorption efficiency can be obtained by very short NWs when following the ARC concept.

Regarding electrical performance, significant improvements have been made possible by innovations like multi-junction/tandem solar cells, back reflectors, the introduction of a back-surface field (BSF) layer, and surface passivation. The latter is a very important technique for NW PV solar cells because NW solar cells that use axial pn junctions tend to experience high levels of surface recombination due to their high surface-to-volume ratio, leading to worse-than-expected electrical performance. Surface passivation can significantly reduce material defects, leading to losses from electron recombination. However, the process of achieving surface passivation may be challenging as well as costly. Another technique for minimizing the possibility of recombination and optimizing the transport of the electron is that of using the radial pn junction rather than the axial pn junction. The radial pn junction has reported excellent results regarding a high PCE since it requires electrons to travel shorter distances, thus minimizing the chances of recombining, which, in turn, leads to the more efficient extraction of electrons [27,35]. In this work, our aim was to analyze the PCE performance of the two-NW and three-NW ARC designs when using a radial pn junction and understand how much the PCE performances of these designs may be impacted by surface recombination. In order to achieve this, we carried out electrical simulations using the finite element method (FEM) [36] that is incorporated into the Lumerical DEVICE Charge package [34].

**Figure 3:**
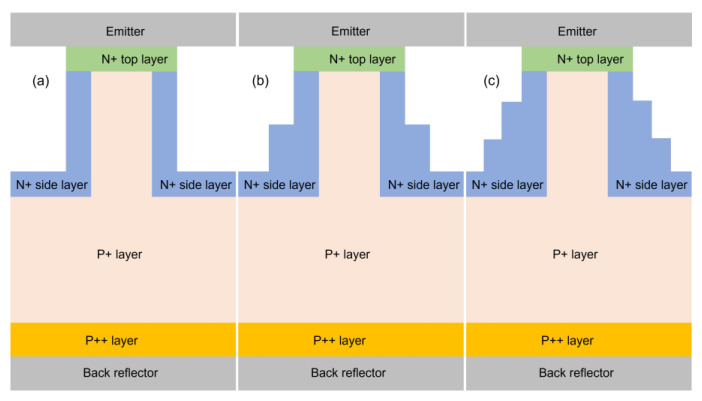
Schematic diagram of the electrical simulations considering a radial pn junction (**a**) for a simple NW, (**b**) for the two-NW ARC design, and (**c**) for the three-NW ARC design.

Figure 3 illustrates the implementation of the radial pn junction technique in our designs for a simple NW and for the two-NW and three-NW ARC designs. Please note that the optical generation file for each of the cases was imported from the corresponding optical code. In all three cases, there is an aluminum layer at the bottom of the simulation window acting as a back reflector as well as the base. Following this, there is a 175 nm-thick p++ layer acting as a BSF layer that is optimized to a p-dopant concentration of 1 × 10^20^ cm^−3^. This is followed by a p+ layer with a p-dopant concentration of 8 × 10^15^ cm^−3^, covering all the structure from just above the p++ layer to the n+ top layer of the NW, bearing only a 150 nm diameter within the NW itself. Subsequently, there is an n+ side with an n-dopant concentration of 2 × 10^17^ cm^−3^, covering all the surrounding walls of the NW and the top part of the substrate. Then, there is a 90 nm-thick n+ top layer covering just the top of the NW. Finally, there is another aluminum layer above the NW acting as an emitter.

**Figure 4:**
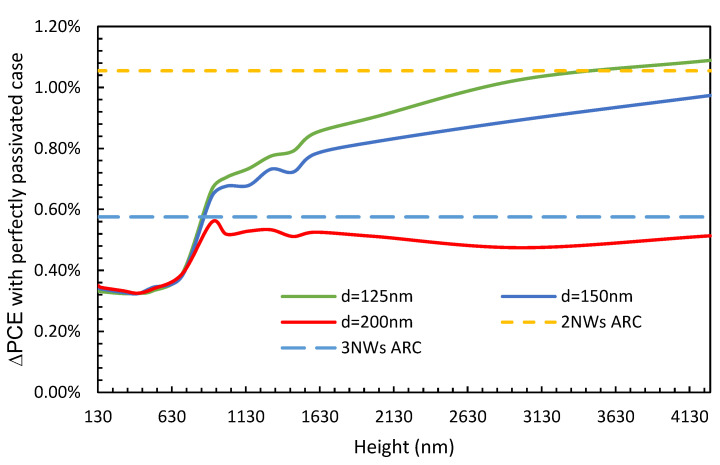
Variations in ∆PCE values between the conventional SRV value and the perfectly passivated case as the height of the NWs increases for various diameters, with the optimized doping profile for the two-NW and three-NW ARC designs.

First, the effect of surface passivation was studied. The structure was solved using a typical surface recombination velocity (SRV) value of 10^7^ cm/s, and the corresponding PCE was compared to that of when the SRV is switched off by setting its value to zero. The variations in the difference in PCE (∆PCE) between these two cases (i.e., the conventional case, where SRV = 10^7^ cm/s, and the perfectly passivated case, where SRV = 0 cm/s) with the height of the NW are shown in Figure 4 for NW diameters of 125 nm (solid green line), 150 nm (solid blue line), and 200 nm (solid red line). The dashed orange line represents the already optimized two-NW ARC design, and the light blue dashed line represents the already optimized three-NW ARC design. From Figure 4, it can be observed that for very short NWs, the impact of surface recombination is very low and increases with height for all diameters under study. In the case of the 200 nm diameter, for a height above 1000 nm, ∆PCE converges and remains at a similar level with further increases in the doping concentration. Please note that, although not shown in Figure 4, similar ∆PCE behavior has been observed when the diameter of the NW is between 175 nm and 275 nm. Furthermore, a very high loss is seen when the diameter is 125 nm, followed by 150 nm, where ∆PCE continuously increases for NWs of heights of as much as 4270 nm. When comparing our two-NW and three-NW ARC designs, it can be observed that the impact in the two-NW ARC case is higher than for diameters of 125 nm and 150 nm at all heights and offers a similar level of impact as diameters of 125 nm for very tall NWs. However, in the case of the three-NW ARC design, it can be observed that ∆PCE is consistently lower than in the cases with a diameter of 125 nm and 150 nm and is very near to the value for the 200 nm case. Please note that we have shown ∆PCE, which is the loss from the surface recombination, and the performance of the actual PCE is shown below.

**Figure 5:**
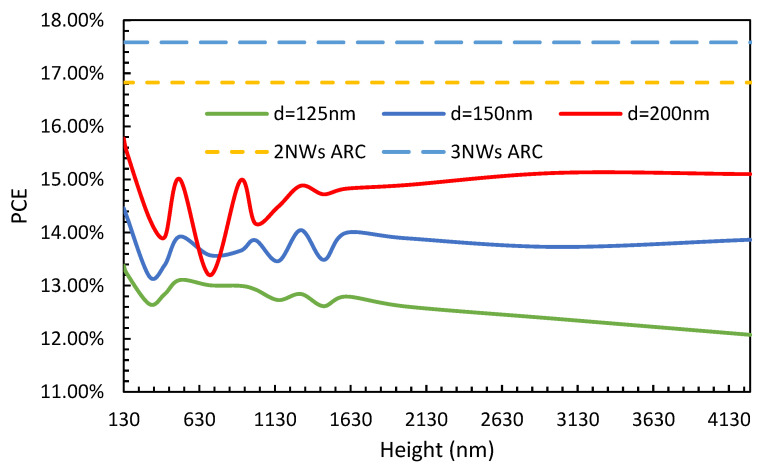
Variations in PCE with height for various diameters, together with the already optimized doping profile for the case of two-NW and three-NW ARC designs, while considering a conventional SRV value.

The variations in PCE for a typical SRV value (at the Silicon-Al interface) of 10^7^ cm/s and the height of the NWs are shown in Figure 5 for NW diameters of 125 nm (solid green line), 150 nm (solid blue line), and 200 nm (solid red line). The dashed orange line represents the already optimized two-NW ARC design, and the light blue dashed line represents the already optimized three-NW ARC design. From Figure 5, it can be observed that the lowest PCE is obtained in the case of the 125 nm diameter, and its PCE did not increase with NW height, which is also highly affected by SRV. The nanowires with a diameter of 150 nm, shown by a blue line, offered slightly better PCE performance, but their poor performance is also affected by SRV. In the 200 nm diameter case, PCE performance was higher than for the other two cases, and its performance is moderately affected by SRV, and the increase in PCE with NW height was very modest. It is not shown here, but for diameters between 175 nm and 225 nm, the corresponding PCE values were very similar to that offered by the 200 nm diameter case. Nevertheless, it can also be observed that the PCE performance of the two-NW and three-NW ARC designs are consistently higher than the other cases. Furthermore, in the case of the three-NW ARC, the PCE is nearly 2% higher than in the case of the two-NW ARC design and is one of the highest reported so far for such a thin-film silicon solar cell with a total pattern height of only 268 nm. Additionally, it is important to highlight that the PCE performance in the case of the three-NW ARC was barely affected by SRV (as shown in Figure 4) when compared to the case of the two-NW ARC.

**Figure 6:**
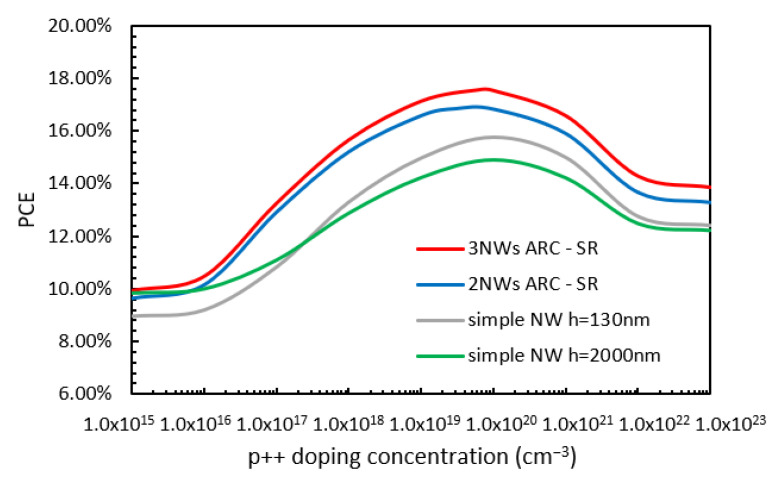
Variations in PCE with p++ layer doping concentration for the already optimized doping profile for the two-NW and three-NW ARC designs.

Next, the effect of various doping concentrations was studied. The variations in PCE with the various doping concentrations of the p++ BSF layers (while all other doping concentrations were kept at their optimized values) for the case of NWs of 130 nm (grey line) and 2000 nm (green line) in height and the cases of the two-NW (blue line) and three-NW (red line) ARC designs are shown in Figure 6. From this figure, it can be observed that for a low p++ layer concentration, the PCE values for the 2000 nm in height, two-NW, and three-NW ARC designs cases are very similar (around 10%), with the PCE value for the 130 nm height case being a few percent below this. Then, as the doping concentration increases, the values for all four cases increase rapidly, reaching their maximum values when the doping concentration reaches 1 × 10^20^ cm^−3^. The maximum PCE values are 17.57%, 16.8%, 15.77%, and 14.9% for the three-NW ARC, two-NW ARC, and NWs with a height of 130 nm and 2000 nm cases, respectively. Then, the PCE value reduces with further increases in the doping concentration until it stabilizes. It should be noted that although a simple NW with a height of 130 nm has a TABS value that is slightly smaller than that of an NW of 2000 nm in height (shown in Figure 2), its overall PCE is actually higher than that of an NW 2000 nm in height due to its better electrical performance, including lower surface passivation loss.

**Figure 7:**
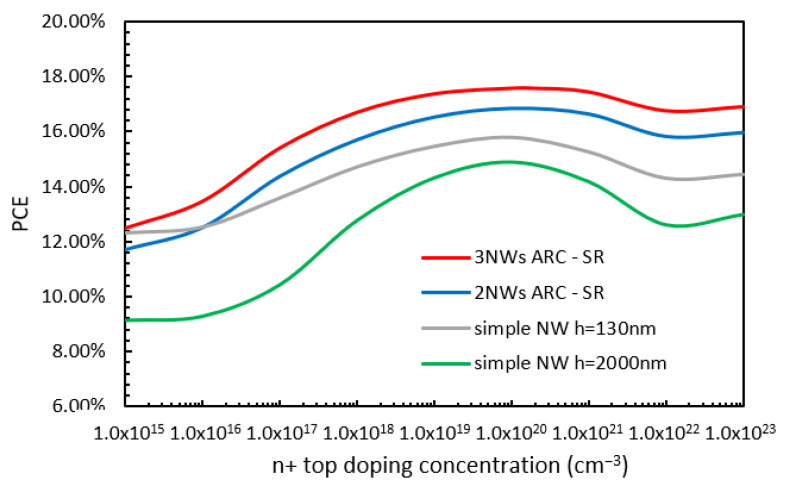
Variations in PCE with n+ top layer doping concentration for the already optimized doping profile for the two-NW and three-NW ARC designs.

Variations in PCE with n+ top layer doping concentration are shown in Figure 7: the NW of height 130 nm (grey line) and 2000 nm (green line), and the cases of two-NW (blue line) and three-NW (red line) ARC designs. Here, all other doping concentrations remained within their optimized values. From Figure 7, it can be seen that for low doping concentrations, the PCE values for the NW 130 nm in height and the two-NW ARC and three-NW ARC designs have similar values to each other (around 12%). In the case of the NW 2000 nm in height, the PCE value is about 2% lower. In all cases, PCE increases with doping concentration (the increase in the case of NW 2000 nm in height seems to be slightly more rapid than in the other cases), reaching their maximum values when the doping concentration is 1 × 10^20^ cm^−3^. The maximum PCE values are 17.57%, 16.8%, 15.77%, and 14.9% for the three-NW ARC, two-NW ARC, and NW 130 nm and 2000 nm in height cases, respectively. For further increases in the level of doping concentration, the PCE continues to increase but at a slower rate.

**Figure 8:**
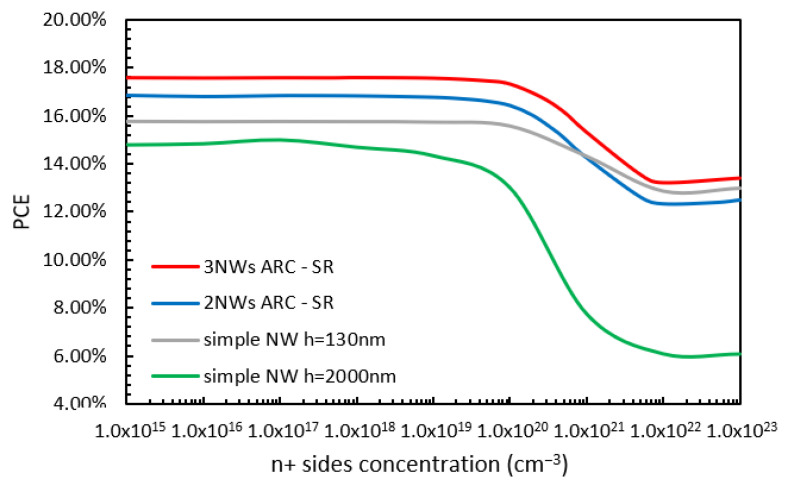
Variations in PCE with n+ sides doping concentration for the already optimized doping profile for the two-NW and three-NW ARC designs.

The variations in PCE with n+ sides layer doping concentration (while all other doping concentrations remained within their optimized values) for the NW 130 nm (grey line) and 2000 nm in height (green line) cases and the two-NW (blue line) and three-NW (red line) ARC designs are shown in Figure 8. From Figure 8, it can be observed that at low doping concentrations, the maximum values of PCE (17.57%, 16.8%, 15.77%, and 14.9%) were obtained for the three-NW ARC, two-NW ARC, NW 130 nm, and 2000 nm in height, respectively. For further increases in the doping concentration, there is no change in the value of PCE until the doping concentration reaches approximately 1 × 10^19^ cm^−3^ for the NW 2000 nm in height case and 1 × 10^20^ cm^−3^ for all others. Thereafter, there is a reduction in the PCE value for all four cases until there is a convergence for any further increase beyond 1 × 10^22^ cm^−3^. 

**Figure 9:**
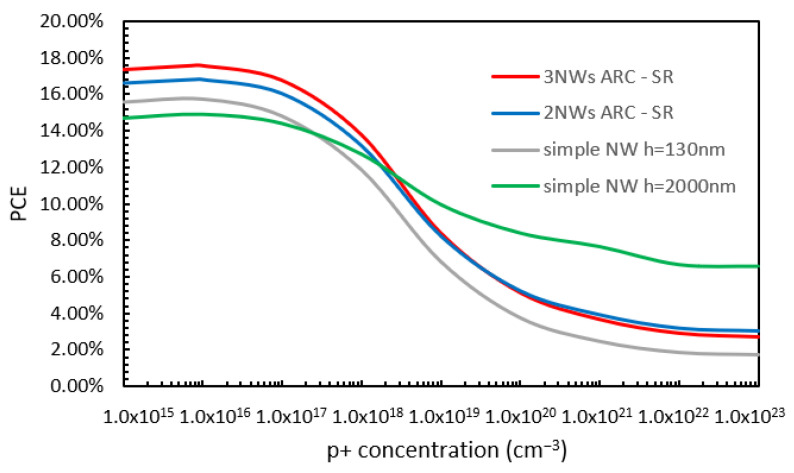
Variations in PCE with p+ layer doping concentration for the already optimized doping profile for the two-NW and three-NW ARC designs.

Finally, variations in PCE with p+ layer doping concentration (while all other doping concentrations remained within their optimized values) for the NW 130 nm (grey line) and 2000 nm in height (green line) cases and the two-NW (blue line) and three-NW (red line) ARC designs are shown in Figure 9. From Figure 9, it can be observed that at low doping concentrations, the values of PCE obtained for the three-NW ARC, two-NW ARC, and NW 130 nm in height cases are significantly higher than for the case of a simple NW with a height of 2000 nm. The maximum values of PCE for the three-NW ARC, two-NW ARC, NW 130 nm, and 2000 nm in height cases (17.57%, 16.8%, 15.77%, and 14.9%) are obtained when the doping concentration reaches 8 × 10^15^ cm^−3^. For further increases in the level of the doping concentration, PCE decreases rapidly at first for all designs, and then it stabilizes when the doping level is increased even further. In the case of the simple NWs with a height of 2000 nm, the decrease in the value of the PCE when the doping level is very high is less significant than for the other designs. However, it is important to highlight the superior performance of the two-NW ARC and the three-NW ARC designs compared to the simple NW designs when the doping concentration levels are within the range of values conventionally reported in the literature for p+ layers. 

## 3. Conclusions

In this paper, we present our concept of mimicking multiple ARC layers to enhance the PCE of solar cells with a radial pn junction. We have carried out a comprehensive analysis via electrical simulations regarding how the PCE performance of our designs is impacted by surface recombination and compared this with the impact experienced by simple NWs of different heights and diameters. For the two-NW ARC design, although the surface recombination loss was higher (due to its much superior optical absorption when compared to simple NWs), the overall PCE was much higher than that of a single NW design as well. Besides this, it was clearly shown that the three-NW ARC design offers the smallest surface recombination loss, and, in addition to this, the corresponding PCE value was also the highest: 17.57%. Furthermore, when comparing the PCE performance of our designs with that of other texturing patterns, such as random inverted pyramidal, and considering a back reflector (below a 20 µm-thick silicon substrate), it was reported a PCE value of 15.5% [37]. Another texturing pattern with which to compare our designs is the quad-crescent-shaped silicon NW pattern, which can generate a PCE of 16.8% when considering NWs that are 2330 nm in height and with radial doping and a back reflector [38]. A moth eye-inspired texturing pattern also drew a lot of attention for its potential to reduce the reflection from the solar cell, but this pattern yields a PCE value of only 9.76% when considering a 700 nm-thick pattern on top of a 10 µm-thick silicon wafer with a back reflector [39]. Therefore, our designs are able to efficiently translate a high level of TABS into the highest PCE value when considering a silicon wafer as thin as 4 µm on top of a back reflector, reducing material usage and, thus, reducing the costs. This is a strong validation of the design since the high PCE performance is not expected to be compromised by surface recombination. Furthermore, we also thoroughly analyzed the PCE performance of our designs against different doping concentrations and compared this with that of NWs of different heights. This is very encouraging since both of our designs consistently offered a much higher PCE than much taller NWs and also showed less sensitivity to the level of the doping concentrations of the n+ top, n+ sides, and p++ BSF layers. In the case of the p+ layer doping concentration, the two-NW ARC and three-NW ARC designs showed higher PCE when considering weakly doped values, which were within the conventionally used values for these types of layers, and the simple NW with a height of 2000 nm showed less sensitivity when considering higher concentrations. For further work, the benefits of this innovative design concept in different aspects might be considered. Adding an additional ARC layer (i.e., 4 NWs ARC design) showed only a slight TABS improvement; thus, the evaluation of their PCE values was not considered here. However, carrying out the optical and electrical optimization of the four-NW and five-NW ARC designs might also be considered in the future. Finally, we can conclude that the present technical advantages of Si-based solar cells over a new generation of alternative solar cells can be maintained by reducing their fabrication costs by using a significantly thinner Si layer, thus adding to their market advantages also.

## Data Availability

The data that support the findings of this study are available upon request from the corresponding author. The data are not publicly available due to privacy or ethical restrictions.
